# Pharmacological fMRI: Effects of subanesthetic ketamine on resting-state functional connectivity in the default mode network, salience network, dorsal attention network and executive control network^☆^

**DOI:** 10.1016/j.nicl.2018.05.037

**Published:** 2018-06-01

**Authors:** Felix Mueller, Francesco Musso, Markus London, Peter de Boer, Norman Zacharias, Georg Winterer

**Affiliations:** a Experimental and Clinical Research Center (ECRC), Dept. of Anesthesiology and Intensive Care Medicine, Charité – University Medicine Berlin, Germany; b Department of Psychiatry, Heinrich-Heine University, Düsseldorf, Germany; c Janssen-Cilag GmbH, Early Development and Clinical Pharmacology, Neuss, Germany; d Janssen Pharmaceutica, Johnson & Johnson Pharmaceutical Research and Development, Beerse, Belgium; e Pharmaimage Biomarker Solutions GmbH, Berlin, Germany; f Pharmaimage Biomarker Solutions Inc., Boston, USA

**Keywords:** Subanesthetic ketamine effects, Cross-over fMRI study, Resting-state fMRI, Seed-to-voxel fMRI analysis, Correlation testing

## Abstract

**Background:**

Subanesthetic dosages of the NMDAR antagonist, S-Ketamine, can cause changes in behavior in healthy subjects, which are similar to the state acute psychosis and are relevant in translational schizophrenia research. Functional magnetic resonance imaging (fMRI) can be used for non-hypothesis-driven analysis of brain connectivity. The correlation between clinical behavioral scores and neuroimaging can help to characterize ketamine effects on healthy brains in resting state.

**Method:**

seventeen healthy, male subjects (mean: 27.42 years, SD: 4.42) were administered an infusion with S-Ketamine (initial bolus 1 mg/kg and continuous infusion of 0.015625 mg/kg/min with dosage reduction −10%/10 min) or saline in a randomized, double-blind, cross-over study. During infusion, resting state connectivity was measured and analyzed with a seed-to-voxel fMRI analysis approach. The seed regions were located in the posterior cingulate cortex, intraparietal sulcus, dorsolateral prefrontal cortex and fronto-insular cortex. Receiver operating characteristics (ROC) were calculated to assess the accuracy of the ketamine-induced functional connectivity changes. Bivariate Pearson correlation was used for correlation testing of functional connectivity changes with changes of clinical scores (PANSS, 5D-ASC).

**Results:**

In the executive network (ECN), ketamine significantly increases the functional connectivity with parts of the anterior cingulum and superior frontal gyrus, but no significant correlations with clinical symptoms were found. Decreased connectivity between the salience network (SN) and the calcarine fissure was found, which is significantly correlated with negative symptoms (PANSS) (R2 > 0.4).

**Conclusion:**

Decreased ketamine-induced functional connectivity in the salience network may qualify as accurate and highly predictive biomarkers for ketamine induced negative symptoms.

## Highlights

• All seed regions showed ketamine induced changes of functional connectivity
• Signficant changes of functional connectivity were found in the salience and executive control network
• PANSS and 5D-ASC scores are highly sensitive and specific to differentiate between placebo and ketamine condition
• A significant correlation between salience network- visual cortex connectivity and negative symptom scores was found

## 1 Introduction

The NMDAR antagonist, *S*-Ketamine, is a widely used anesthetic and analgesic agent in clinical practice (Niesters et al., 2012). In sub-anesthetic dosages, its effects on the central nervous system mimic positive and negative symptoms in healthy subjects similar to acute psychosis or schizophrenia while also exacerbating clinical symptoms in schizophrenia patients (Lahti et al., 2001; Frohlich and Van Horn, 2014). Therefore, ketamine is generally considered to be a useful pharmacological model for glutamatergic dysfunction in schizophrenia. Investigating ketamine-dependent changes of brain connectivity is a promising approach to understand the underlying mechanisms of these clinical symptoms. Furthermore, subanesthetic ketamine is a promising medication for treatment resistant depression (Berman et al., 2000), but it's still unknown, whether psychotomimetic side effects can be fully segregated from the therapeutic effect (Maltbie et al., 2017). Ketamine-induced changes of functional connectivity might be used as a surrogate outcome measure (functional biomarker) when conducting drug trials to assess the dose-reponse relationship of psychotomimetic ketamine effects. In an ideal scenario, the combination of imaging markers and clinical parameters could be used to find dosages that minimize the positive/negative symptoms while sufficiently treating patients, e.g. with major depression.

In order to investigate ketamine effects on the brain a widely used non-invasive approach to measure regional and global brain connectivity with high spatial and temporal resolution is functional magnetic resonance imaging (fMRI). For example, fMRI can measure regional brain activity indirectly by fluctuations in the blood‑oxygen-level dependent (BOLD) signal within the brain vessels during an MR-scan (Buxton, 2013). Recent studies suggest that neural activity and perfusion stay coupled during subanesthetic ketamine infusion (Maltbie et al., 2017).

Non-task related brain activity can be measured by using resting-state fMRI (RS-fMRI) and connectivity analysis can reveal intrinsic resting state networks, which show coherent fluctuations of BOLD-signal. This method allows non-a priori hypothesis driven analysis of the brain activity (Niesters et al., 2012). Recent fMRI research on ketamine uses mainly two different analysis approaches: global brain connectivity and seed-based analysis (Maltbie et al., 2017).

In global connectivity analysis an overall increase of resting-state functional connectivity across widely distributed networks has been reported for acute drug challenge (Driesen et al., 2013). This study showed a correlation between positive schizophrenia symptom scores and increased global connectivity in ketamine. Another study on global connectivity by Joules et al, demonstrated overall decreased cortical and increased subcortical functional connectivity (Joules et al., 2015).

Effects of sub-anesthetic ketamine administration on RS-fMRI functional connectivity in healthy subjects were extensively investigated by recent research using Region of Interest (ROI)-analyses (Scheidegger et al., 2016; Grimm et al., 2015; Khalili-Mahani et al., 2015; Niesters et al., 2012; Scheidegger et al., 2012). These experiments differed in dosage of administered ketamine, ranging from 0.25 mg/kg body weight (Scheidegger et al., 2012) to 0.57 mg/kg (Niesters et al., 2012; Khalili-Mahani et al., 2015). Another difference between the experiments was the period of RS-fMRI data acquisition, ranging from simultaneous infusion and scan (e.g. Khalili-Mahani et al., 2015) to control scans 24 h after infusion (Scheidegger et al., 2012). In contrast to these studies, a different approach was chosen by Bonhomme et al… by studying ketamine resting-state effects in different clinical sedation levels of their subjects without a predefined ketamine plasma levels (Bonhomme et al., 2016).

Acute ketamine challenge increases positive connectivity between the hippocampus and the dorsolateral prefrontal cortex (DLPFC) (Grimm et al., 2015). In another study, increased connectivity was found between the hippocampus with cingulate, precuneal, cerebellar and basal ganglia regions (Khalili-Mahani et al., 2015). By using an amygdala seed an increased connectivity with the precuneal anterior cingulate was reported (Scheidegger et al., 2016). Niesters et al selected cortical seed regions only and showed increased functional connectivity between the medial visual network with cerebellum and visual cortex. Additionally, decreased connectivity was shown between the auditory and somatosensory cortex on one hand and the amygdala, anterior cingulate cortex (ACC) and insula on the other hand (Niesters et al., 2012). Within the default mode network (DMN), an overall decreased connectivity was found 24 h after ketamine infusion between its representative hub, the posterior cingulate cortex (PCC) and the medial prefrontal cortex and ACC (Scheidegger et al., 2012). By using a target-controlled infusion approach with the Domino model, Bonhomme et al reached different levels of sedation in their subjects from light sedation to unresponsiveness (Bonhomme et al., 2016). Resting-state functional connectivity in ROIs of the DMN, ECN, SN, auditory network, sensorimotor network and visual network was compared in these different responsiveness states. It was shown that deeper sedation decreased DMN functional connectivity, especially with the prefrontal cortex and anticorrelated activity between the DMN and other brain regions was significantly reduced as well. SN functional connectivity was decreased by ketamine, but nonuniformly between subjects. The ECN functional connectivity was minimally affected in the experiment.

Here, we conducted a double-blind, randomized, placebo-controlled cross-over subanesthetic ketamine-drug challenge experiment in healthy male subjects. It was the primary aim of this study to answer the question whether ketamine-induced effects on functional connectivity in resting state networks potentially qualifies as a surrogate outcome measure (functional biomarker) to be used in research of ketamine side effects, for instance in dosage-response studies of subanesthetic ketamine. We investigated ketamine effects on RS-fMRI, assessed its accuracy to predict drug effects and correlated drug effects on functional connectivity with clinical symptom changes before and after each scan session. For assessing symptom changes, we used the Positive and Negative Symptom Scale of Schizophrenia (PANSS) (Kay et al., 1987) and - for exploratory reasons - the Altered State of Consciousness (5D-ASC) Rating Scale (Dittrich, 1998). We selected functional networks for a seed-to-voxel analysis that have been previously implicated in schizophrenia (Woodward et al., 2011): the DMN, the dorsal attention network (DAN), the executive control network (ECN) and the salience network (SN) which are associated with memory, attention and executive cognitive functions. These functional networks were identified as “higher functional networks” by recent research (Woodward et al., 2011). The linkage between schizophrenia and ketamine effects is particularly interesting for our study. Therefore, we aim to compare both studies and focus exclusively on these large resting state networks.

## 2 Methods and materials

The study was conducted in compliance with the Ethical principles of the WMA Declaration of Helsinki, the EU Clinical Trial Directive 2001/20/EC and the German Medicines Act (Arzneimittelgesetz). Approval was obtained by the local ethics committee (Ärztekammer Nordrhein, Düsseldorf, Germany) and by the German federal drug agency (Bundesinstitut für Arzneimittel und Medizinprodukte, BfArM). As a Phase-I study, this clinical trial was registered in the non-public European Union Drug Regulating Authorities Clinical Trials (EudraCT) repository.

### 2.1 Participants

As outlined in more detail elsewhere (Musso et al., 2011), male volunteers were recruited via newspapers and the internet. Prior to study inclusion, full written consent was obtained and a medical, neurological and psychiatric examination (including assessment of Diagnostic and Statistical Manual of Mental Disorders (DSM)-IV Axis I/II diagnoses) by a certified neurologist and psychiatrist were performed. Twenty-four healthy male subjects participated in the study. Male subjects were recruited exclusively to avoid additional screenings, e.g. pregnancy tests, prior to the study. Research on functional connectivity between interhemispheric corresponding brain regions in fMRI suggests between-gender differences in resting state functional connectivity, especially in the prefrontal cortex and amygdala Zuo et al., 2010). While these differences would have been particularly interesting for our study regarding ketamine effects, adding functional connectivity variance to an analysis with a limited number of subjects, e.g. through between-gender differences, was a major concern, possibly reducing the sensitivity to ketamine effects.

Two subjects dropped out because of adverse events during first scan session (panic attack, tachycardia), one was administered ketamine and the other one was administered placebo. N = 5 subjects were excluded from further analysis, because at least one RS-fMRI session wasn't acquired. These subjects showed excessive head motion due to positive symptoms (excitement, hallucinations…) during the ketamine sessions, which therefore prematurely terminated by the study conductors. Subjects with prematurely terminated RS-fMRI scans weren't excluded from further analysis. Overall, seventeen subjects (mean age: 27.42 years, age range: 21–35 years, SD: 4.42) were included in this analysis. All subjects except one were right handed. *Exclusion criteria* were: age below 18 years or over 35 years, past medical history of a medical, neurological, psychiatric or ophthalmic disease, drug medication less than four weeks before the trial, history of drug abuse, first or second degree relatives with schizophrenia, clinically relevant abnormalities in blood/drug test, claustrophobia, metal implants (prosthesis, cardiac pacemakers), missing written informed consent, excessive head motion during RS-fMRI echo planar imaging (EPI) session (x, y or z-translation >3 mm compared to mean) and missing RS-fMRI sessions.

### 2.2 Study design

This study was a randomized, double-blind, placebo-controlled cross-over trial. On two separate scan sessions, the subjects were either challenged with *S*-ketamine (*Ketanest S*, Pfizer Pharma PFE, Berlin, Germany) and saline (0.9% NaCl) intravenously right before the start and during the MR scan. There was at least a one week between the two MRI scan sessions for each subject to avoid residual drug effects on the cross-over scan. The order of ketamine or placebo sessions was randomly assigned and counterbalanced. Upon arrival, subjects received a psychopathological evaluation, which was performed by a psychiatrist by using PANSS and 5D-ASC. The PANSS includes the following items: “positive symptoms”, “negative symptoms” and “sum”. “Positive symptoms” are defined by scale items like delusions, conceptional disorganization, hallucinatory behavior, excitement, grandiosity, suspiciousness and hostility (Kay et al., 1987). “Negative symptoms” include blunted affect, emotional withdrawal, poor rapport, passive-apathetic social withdrawal, difficulties in abstract thinking, lack of spontaneity and stereotypical thinking (Kay et al., 1987). The 5D-ASC was tested with the following subitems: “oceanic boundlessness” (OBN, referring to dissolution of ego boundaries associated with positive emotions), “dread of ego dissolution” (DED, distressing experience of depersonalization, thought disorder and loss of body control), “visionary restructuralization” (VRS, e.g. visual illusions and hallucinations), “auditory alterations” (AUA, e.g. illusions and hallucinations) and “vigilance reduction” (VIR) (Studerus et al., 2010). This evaluation was repeated directly after the MR scan. The subjects received an intravenous line and were transferred to the MR scanner. A bolus of 0.1 mg/kg *S*- ketamine (or equal volumes of saline) was administered, then the infusion stopped for one minute after MR scan initiation and continued during the scan with 0.015625 mg/kg/min (max. one hour) with a dosage reduction of 10% every 10 min. This was performed in order to maintain stable ketamine plasma levels during the experiment as ketamine slowly accumulates during infusion (Feng et al., 1995). Ketamine plasma levels were not measured during this experiment. But the infusion regime was chosen to reach relatively stable plasma levels and is derived from an earlier ketamine study (Umbricht et al., 2000). By using the STANPUMP simulation software and the domino model for ketamine, our infusion regimen was tested exemplarily for a twenty-five years old male subject with 180 cm height and 80 kg weight (Shafer, 2008). Using a fixed infusion rate of 0.02 mg/kg/min, which was used due to the limited digit input of the software, plasma levels for ketamine were 0.4 μg/ml after one minute, lowest after five minutes with 0.25 μg/ml and were slowly increasing to 0.48 μg/ml after 90 min (longest period of infusion). Therefore, we can assume that the plasma levels were between 0.25 μg/ml and lower than 0.48 μg/ml during the whole experiment. The intravenous infusion was monitored by a board-certified anesthetist. Additionally, vital signs (blood pressure, peripheral *p*O_2_-saturation and partial CO_2_-pressure) were monitored but weren't recorded synchronously with the fMRI sessions and weren't available for correlation testing in this analysis.

### 2.3 fMRI data acquisition

**Fig. 1:**
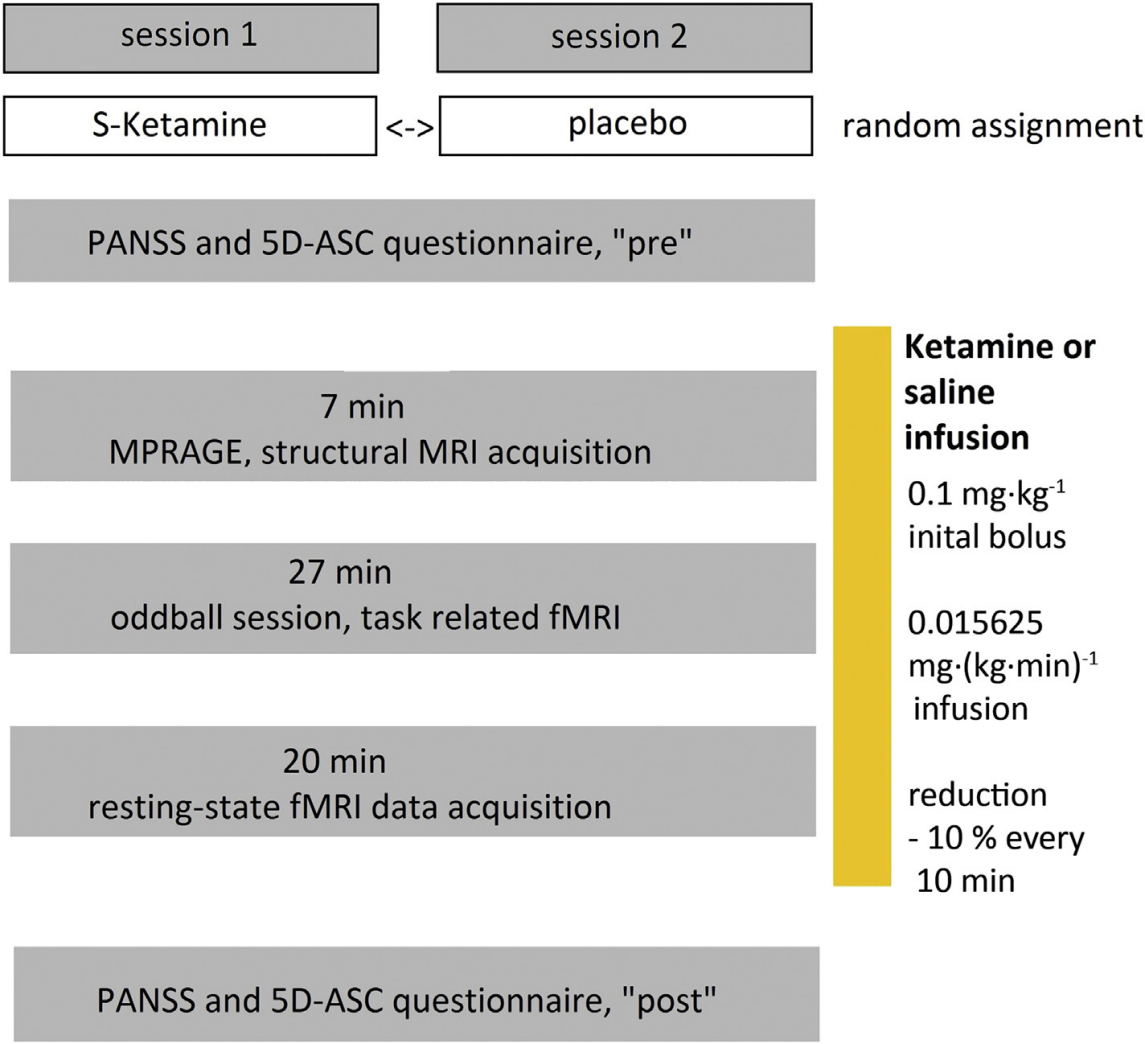
Study Procedures: Seventeen subjects were randomly assigned to either ketamine or placebo (saline) infusion (cross-over: >one week apart).

The MR scans were performed using a 3 T MR Scanner (Trio, Siemens, Erlangen, Germany). The MRI scanning sequence consisted of a structural MR scan during minute 0–7, a task-related MR sequence using a visual oddball task (checkerboard reversal) from minute 8 to 34 (already published by Musso et al., 2011) and a resting-state EPI session of 20 min which is presented in the present paper (see Fig. 1). The structural images were acquired by using the following settings: T1-weighted MR sequence (magnetization-prepared rapid gradient-echo sequence): repetition time/echo time = 2250/3.03 ms, flip angle = 9°, 176 sagittal slices, FOV 200 × 200 mm, 64 × 64 matrix, voxel size 1 × 1 × 1 mm. The following MRI sequence parameters were used in the EPI sessions (RS-fMRI): 33 slices; slice order: ascending, slice thickness: 3 mm, field of view (FOV) 200 × 200 mm, 64 × 64 matrix, voxel size: 3 × 3 × 3 mm, repetition time 3400 ms, echo time 40 ms, flip angle 90°.

### 2.4 fMRI data processing

Prematurely terminated scan sessions during ketamine challenge required shortening all scan sessions to the same length in order to compare data between subjects. This approach was chosen to include subjects showing more positive symptoms (e.g. excitement, hallucinations) as well as less affected subjects equally without causing a selection bias by excluding subjects with prematurely terminated scans during drug challenge. Furthermore, shorter sessions could potentially reduce the influence of head motion on further analysis. The numbers of all acquired RS-fMRI volumes for each condition are shown in Table 1 with the shortest ketamine scan consisting of 163 volumes. All data were shortened to the first 150 volumes (duration = 8 min 30 s) for further analysis, excluding the first five volumes from each session to minimize the influence of technical artifacts of the MRI scanner (FEAT User guide, 2016). The overall analyzed resting-state period is within the standard range of recent studies (3-11 min) (Birn et al., 2013). Shortening the sessions to the same length was performed to avoid different resting-state periods as an additional source of confounds. The functional connectivity is calculated from signal correlation between seed region and target area over a time course. Not only could shortening or extending the time course between subjects cause a selection bias, e.g. favoring more stable normal resting-state connectivity over short instable ketamine effects in a less affected subject, but add other between-subject confounds, e.g. vigilance reduction during longer periods with closed eyes.

**Table 1:** Overview on all acquired resting state fMRI volumes of included subjects (N = 17).

Subject number	Placebo session	Ketamine session
	(number of volumes)	(number of volumes)
1	400	400
2	400	400
3	400	202
4	400	204
5	400	320
6	400	315
7	400	199
8	400	290
9	400	163
10	400	200
11	400	400
12	400	400
13	315	320
14	400	241
15	400	200
16	400	302
17	400	400
Shortest session	315	163
Mean session length	395	291.53
Paired *t*-test	p-Value	0.000249693

To compare the head motion of the included volumes between ketamine and placebo, the six realignment parameters for translation and rotation from the latter described fMRI preprocessing were tested for significant differences of mean head motion and mean head rotation. Mean head motion was calculated as mean value of all head displacements per subject and session (displacement = square root (x^2^ + y^2^ + z^2^)) and mean head rotation as the mean of all absolute Euler angles for each subject and session (Euler angle = arccos((cos(pitch)cos(roll) + cos(pitch)cos(yaw) + cos(roll)cos(yaw) + sin(pitch)(sin(yaw)sin(roll) - 1)/2)) (Van Dijk et al., 2012).

Functional EPI data were preprocessed using *Statistical Parametric Mapping* *12* (*SPM 12*, Wellcome Trust, London, UK). The following steps were performed: 1) slice timing correction, 2) spatial realignment to mean with rigid body correction (three translation and three rotation parameters), testing for excessive head motion or rotation in any direction (>3 mm or 3°), 3) coregistration of the structural image and the functional data, 4) segmentation of brain tissues (grey matter, white matter and cerebrospinal fluid (CSF)), 5) spatial normalization to standard Montreal Neurological Institute (MNI) space and 6) smoothing with full width at half maximum (FWHM) = 8 × 8 × 8 mm kernel.

### 2.5 FMRI analysis and ROC curves

Preprocessed data were analyzed by using the *CONN connectivity toolbox V15* (Gabrieli Lab, Massachusetts, USA). Experimental data of all subjects were sorted into two groups (placebo or ketamine). For reduction of motion related artifacts, session specific realignment parameters from preprocessing were used as first level covariates. Reduction of confounding noise signals was performed with *CONN* standard processing steps including regression of CSF and white matter BOLD-signals, despiking, nuisance regression of motion related components via component-based noise correction method (CompCor), linear detrending and band-pass filtering (0.008 to 0.09 Hz) before connectivity analysis (Whitfield-Gabrieli and Nieto-Castanon, 2012). Further correction of micro motion, e.g. by using scrubbing or wavelet despiking, was not performed.

For seed-to-voxel analysis the seed ROIs were defined as 6 mm radius spheres around MNI coordinates. The ROI volume size was chosen accordingly to the values used in the publication by Woodward et al. At a voxel size of 3x3x3mm, the 6 mm radius of the spherical ROIs is a compromise between avoiding overlapping between functionally distinct brain regions in a single seed region and including multiple voxels in a ROI to represent cluster-wise activation rather than single voxel activation. The ROIs are located in the following brain regions: PCC (1–55 17) for the DMN, left and right intraparietal sulcus (−25–53 52 / 25–57 52) for DAN, left and right DLPFC (−42 34 20 / 44 36 20) for ECN and left and right fronto-insular cortex (−32 26–14 / 38 22 10) for SN (Woodward et al., 2011). For seed-to-voxel analysis, a seed mask was created of each functional network including the ROIs for left and right hemisphere. On single level for each ROI the mean BOLD-signal time course was extracted, tested for correlation with every other voxel in the brain using bivariate Pearson correlation. The correlation coefficients were then Fisher r-to-z transformed and used to create z-score correlation maps. On these maps second level voxel-wise statistics was performed in *CONN,* using a general linear model. Several sources of variance were removed by linear regression (e.g. white matter, CSF signals) and two-sided between condition contrasts using paired-*t*-tests were performed. For these voxel-wise statistics the following thresholds were applied: False-discovery rate (FDR) corrected cluster extent threshold p-value<0.05 and “peak-level” uncorrected height threshold p-value <0.001. These thresholds are sufficient for correction of multiple comparisons (CONN FAQ, 2016). Resulting clusters of interest are visually presented using *BrainNet viewer* (Beijing Normal University, Beijing, China). This fMRI data analysis is performed on one data set, but for four different seed regions separately. Therefore, an increased family-wise error rate is a major concern. By lowering the cluster extent threshold after FDR-correction, from p < 0.05 to p-value <0.0125 (Bonferroni correction, 0.05 *α*-value divided by number of tested seed regions) for further analysis, the problem of multiple testing is addressed. The first level z-scores of significant clusters of interest from second level analysis with p < 0.0125 are extracted from *CONN* for every subject and condition. Further statistical analysis was performed using *SPSS* (IBM Corp., Armonk, NY, USA). Delta values are calculated by subtraction of placebo z-scores from ketamine z-scores (ketamine- placebo).

Receiver Operating Characteristic (ROC) curves were calculated for these delta values to test their specificity and sensitivity to differentiate placebo and ketamine condition.

### 2.6 Correlation testing with symptom scores

The PANSS and 5D ASC subitems were tested before and after every scan session; therefore, delta values were calculated to represent change of each subitem during the scan (postsession- presession value). The PANSS and 5D-ASC subitems were tested for significant differences in ketamine condition by using paired *t*-tests. For each subject, delta values comparing both conditions (ketamine session - placebo session) were calculated. Each delta value of a significant fMRI signal change in first level analysis was tested for correlation with the according delta value of a clinical score by using bivariate Pearson two-tailed correlation p < 0.00625 (eight tests, p-level was adjusted for multiple comparison with Bonferroni correction). Additionally, correlation testing between the delta of session lengths (ketamine- placebo) and delta clinical scores (ketamine- placebo) was performed to rule out prematurely terminated sessions as significant source of confounds.

## 3 Results

### 3.1 Head motion

**Fig. 2:**
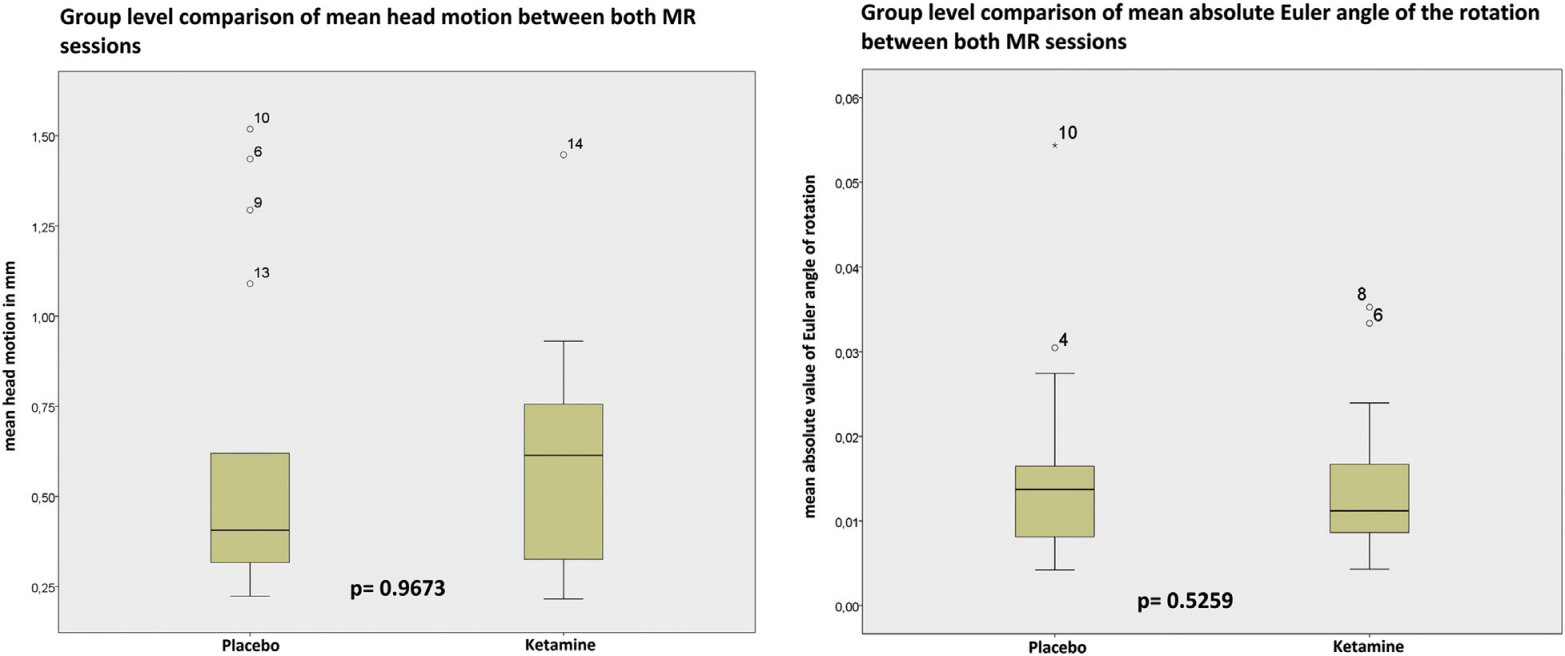
Boxplots for group mean values of head motion and head rotation compared between both conditions. P-value of paired t-test between groups indicates no significant differences of mean values. (N = 17).

In Table 2, Table 3 mean head motion and mean absolute Euler angles are represented, the distribution of values is visualized using boxplots in Fig. 2. A group-level paired t-test for mean head motion and mean rotation showed no significant difference between ketamine and placebo on group level (head motion p-value = 0.967 and rotation p-value = 0.526).

**Table 2:** Mean motion of subjects (N = 17) calculated as mean of all displacements according to realignment parameters (displacement = square root (x^2^ + y^2^ + z^2^)) in mm.

Subject no.	Placebo	Ketamine
1	0.317	0.637
2	0.619	0.393
3	0.416	0.930
4	0.336	0.264
5	0.295	0.325
6	1.435	0.614
7	0.234	0.285
8	0.223	0.756
9	1.294	0.455
10	1.518	0.900
11	0.316	0.216
12	0.422	0.756
13	1.089	0.499
14	0.327	1.447
15	0.406	0.256
16	0.382	0.700
17	0.543	0.651
Mean value	0.598	0.593
Paired *t*-test	p-Value	0.967

**Table 3:** Mean Euler angle of rotation of subjects (N = 17) calculated as mean of all absolute Euler angles (Euler angle = arccos((cos(pitch)cos(roll) + cos(pitch)cos(yaw) + cos(roll)cos(yaw) + sin(pitch)sin(yaw)sin(roll) − 1)/2)).

Subject no.	Placebo	Ketamine
1	0.016	0.007
2	0.017	0.009
3	0.014	0.012
4	0.030	0.010
5	0.013	0.011
6	0.027	0.033
7	0.005	0.005
8	0.004	0.035
9	0.016	0.011
10	0.054	0.011
11	0.015	0.009
12	0.012	0.017
13	0.014	0.004
14	0.005	0.017
15	0.016	0.005
16	0.008	0.011
17	0.007	0.024
Average	0.016	0.014
Paired *t*-test	p-Value	0.526

### 3.2 Ketamine effects on resting state functional networks

**Fig. 3:**
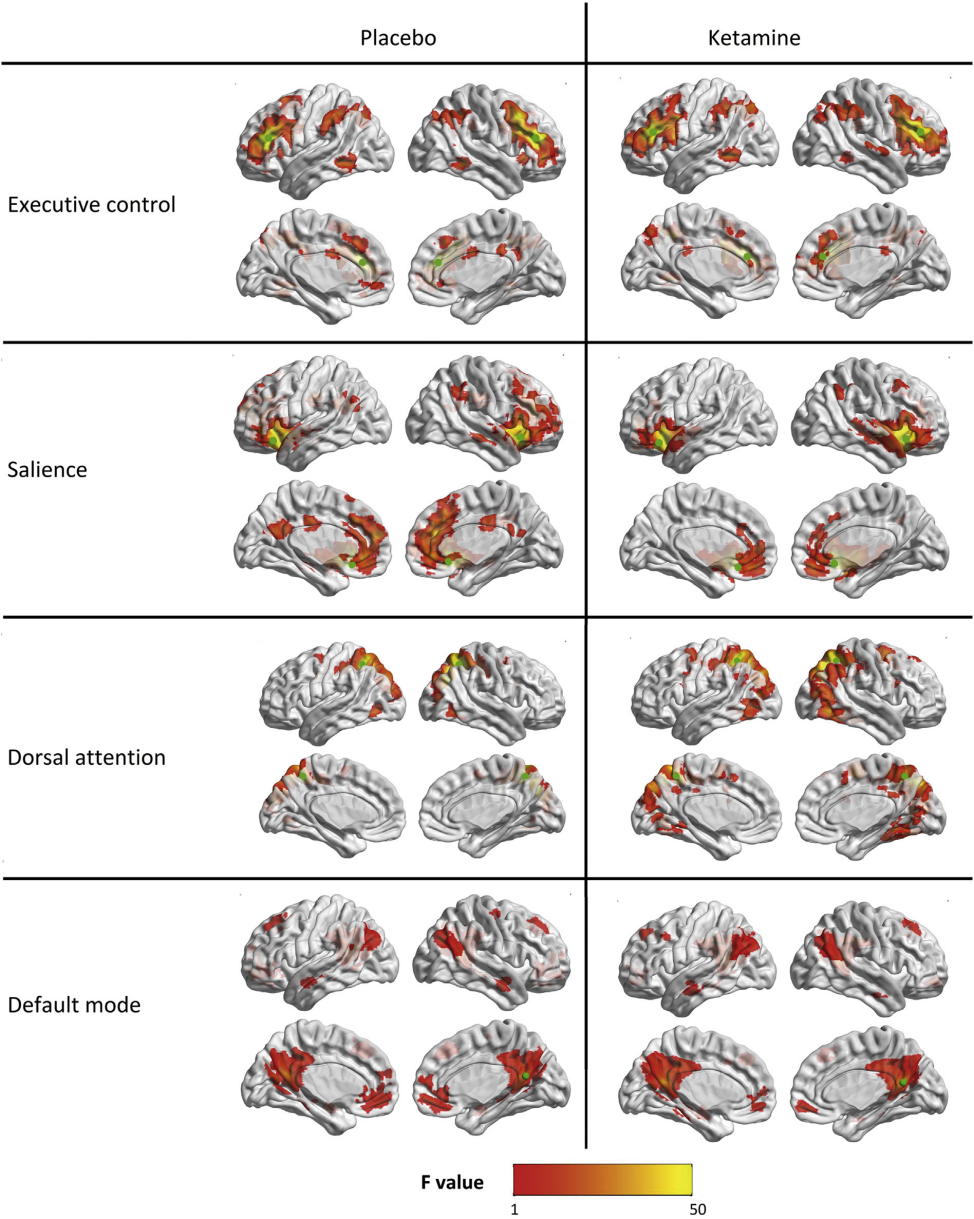
Separate ketamine and placebo effects in N = 17 healthy subjects. Shown are positive effects (positive signal correlation of BOLD signal between seed regions and other brain voxels) with second level result maps of seed-to-voxel analyses for all four networks: ECN, SN, DAN and DMN. The seed regions of each network are indicated as green circles. Significant results without additional Bonferroni correction of thresholds. No negative ketamine effects (negative signal correlation of BOLD signals) were observed. (For interpretation of the references to color in this figure legend, the reader is referred to the web version of this article.)

**Fig. 4:**
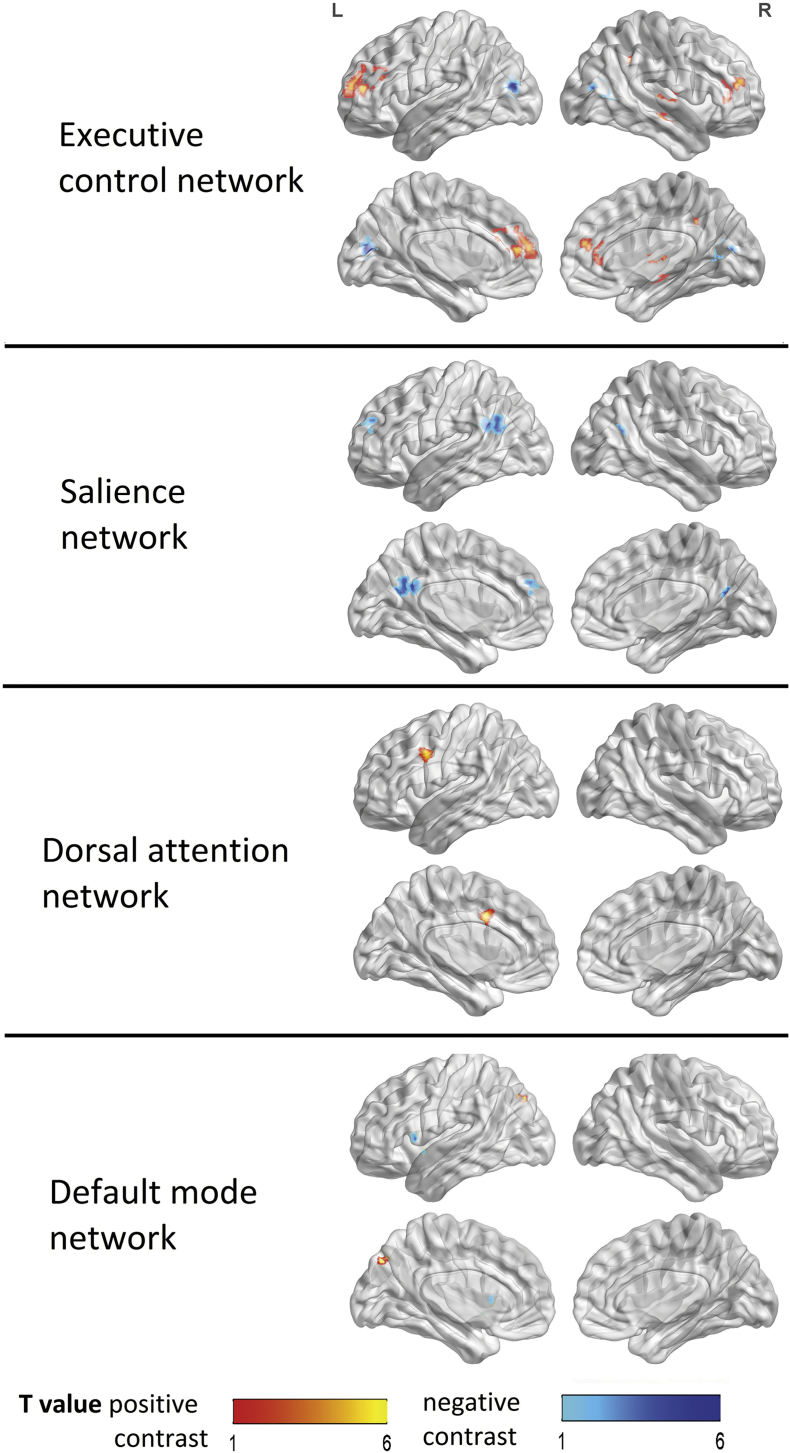
Between conditions second level contrast maps with significant results without Bonferroni correction applied. Positive contrasts (ketamine> placebo) are shown in red colors and negative contrasts (placebo> ketamine) are shown in blue for each seed region in the same figure. (Note: SN only showed significant negative contrasts and DAN only positive contrasts; see Table 1). (N = 17). (For interpretation of the references to color in this figure legend, the reader is referred to the web version of this article.)

**Fig. 5:**
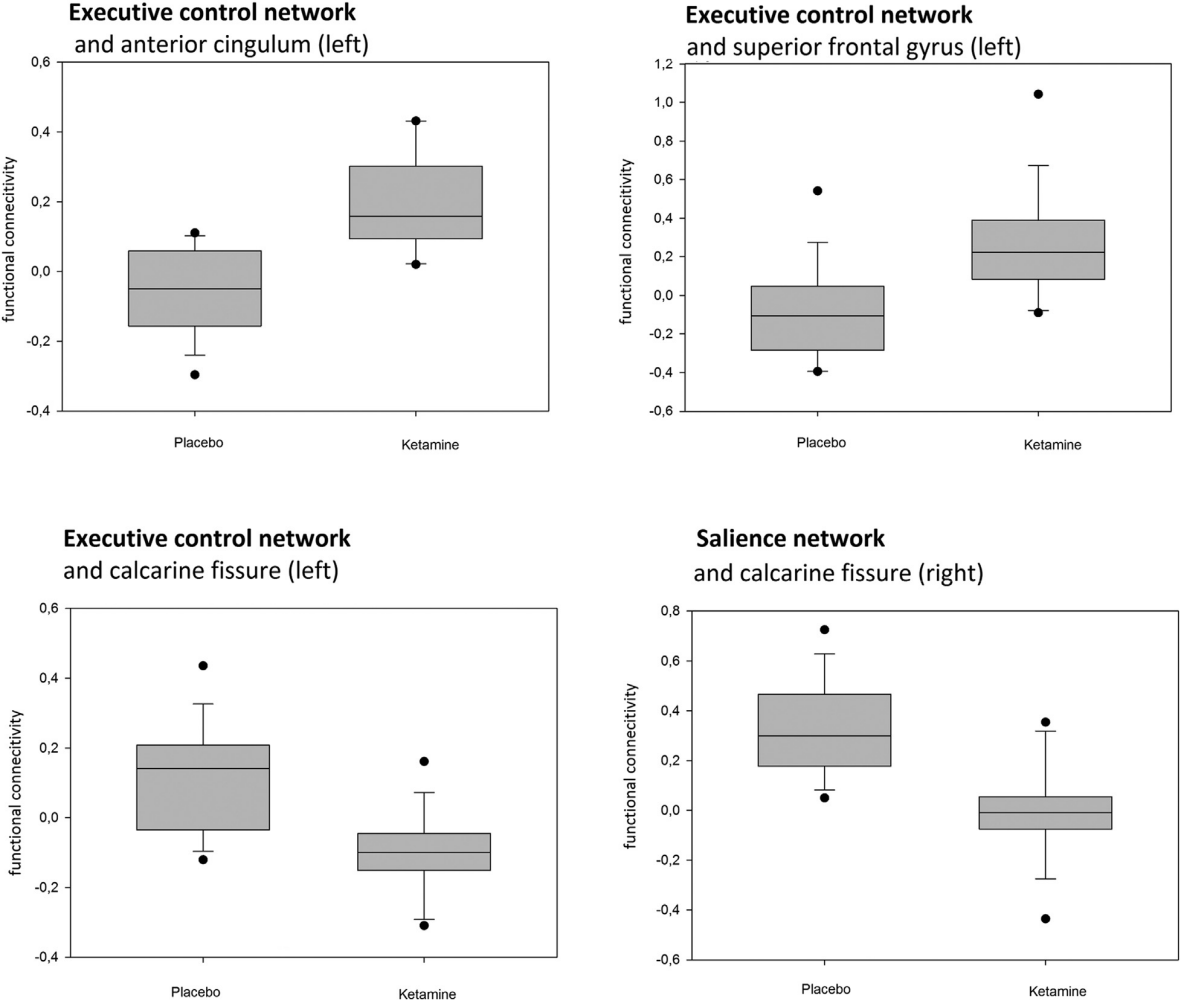
Ketamine vs. placebo condition (N = 17 subjects, cross-over): Boxplots of single level results for the all contrasts of second level analysis that are significant after Bonferroni correction. Functional connectivity (delta Fisher's *Z*-score (ketamine- placebo)). (For interpretation of the references to color in this figure legend, the reader is referred to the web version of this article.)

**Fig. 6:**
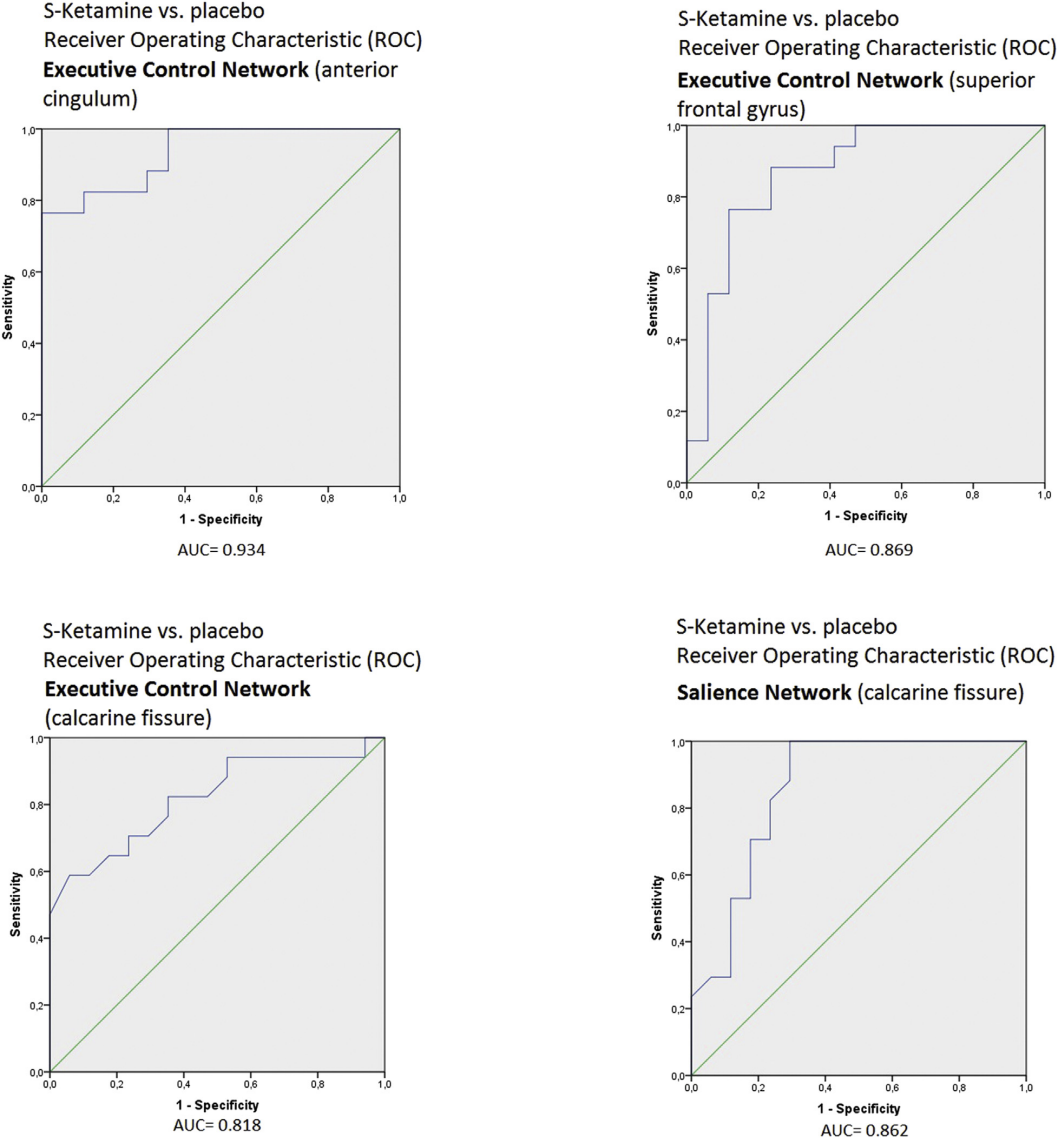
ROC curves for significant contrasts of second level analysis (after Bonferroni correction). Functional connectivity (delta Fisher's z-score) is used as parameter to differentiate between ketamine and placebo condition.

Second level correlation maps with no Bonferroni correction applied are shown for each condition and seed region in Fig. 3. Only clusters with positive correlations with the seed regions were found. Two-sided contrast maps without Bonferroni corrected are shown in Fig. 4 and Table 4. Increased correlation in the ketamine condition was shown for the DLPFC (ECN) with Left (L.) anterior cingulum, L. superior frontal gyrus, Right (R.) medial temporal lobe, R. angular gyrus and R. superior temporal lobe. Decreased correlation was found with the calcarine fissure bilaterally. The increased correlation with L. anterior cingulum, L. superior frontal gyrus and the decreased correlation with L. calcarine fissure survived Bonferroni correction. No increased correlation in ketamine condition was found for the fronto-insular cortex (SN), but decreased correlation with the R. calcarine fissure (this survives Bonferroni correction) and L. frontal gyrus. The infraparietal sulcus (DAN) showed increased correlation with the L. precentral gyrus, but no decreased correlation in ketamine condition. The posterior cingulate cortex (DMN) showed increased correlation with the superior parietal lobule and decreased correlation with the L. Rolandic operculum in ketamine condition. For the results, which survived Bonferroni correction boxplots showing differences of delta z-scores on group level between conditions are shown in Fig. 5. For the four correlations, which survive Bonferroni correction ROC curves were calculated presented in Fig. 6. For this specific experiment changes of the fMRI correlations were highly specific and sensitive to differentiate between ketamine and placebo condition with Area under the Curve (AUC) = 0.934 for ECN-anterior cingulum, 0.869 for ECN- superior frontal gyrus, 0.818 for ECN- calcarine fissure and 0.862 SN- calcarine fissure. The increased correlations due to Ketamine were slightly more specific and sensitive than the negative correlations.

**Table 4:**
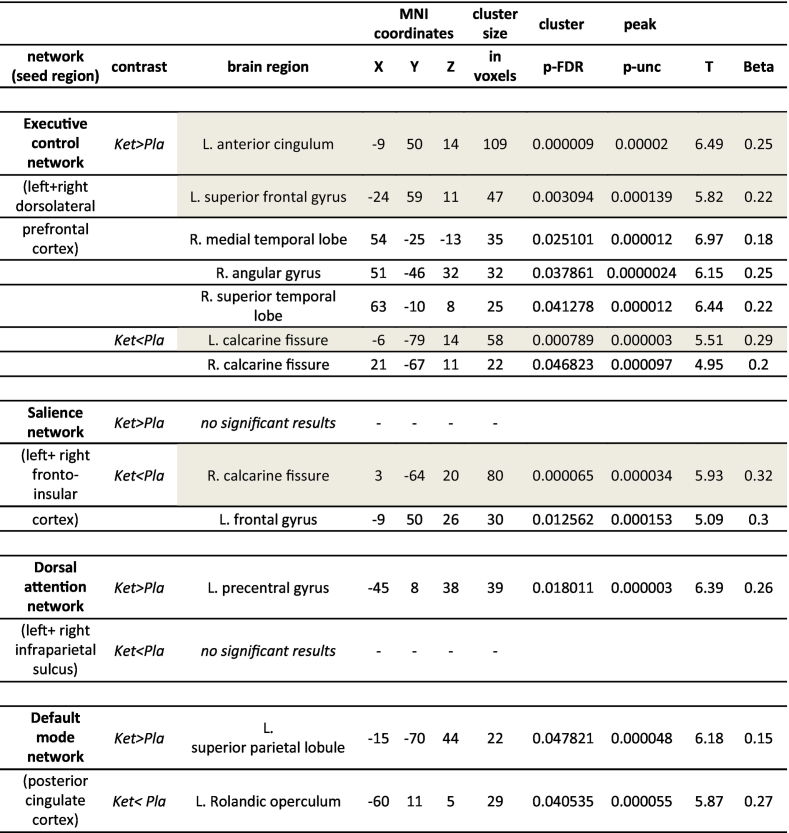
Resting-state network alteration comparison between ketamine and placebo condition; Bonferroni correction not applied.

Grey background indicates significant contrasts after Bonferroni correction. Ket>Pla contrast represents Ketamine over Placebo between condition contrast calculated with paired t-test (p < 0.05). Ket< Pla contrast represents Placebo over Ketamine contrast in second level analysis.

Abbreviations: Cluster p-FDR (cluster level p-value false discovery rate corrected), peak p-unc (peak level p-value uncorrected), T (t-value of one sample *t*-test), Beta (fisher transformed correlation coefficients between seed and target area).

### 3.3 Correlation testing with symptom scores

**Fig. 7:**
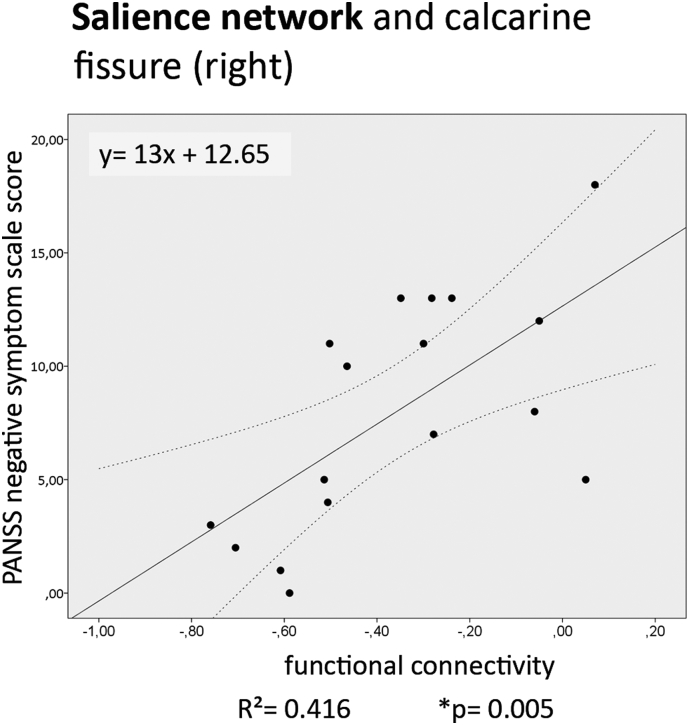
Correlation testing between clinical symptoms and functional connectivity: Significant correlation between the PANSS negative symptom delta score (ketamine- placebo) and the SN- R. calcarine fissure functional connectivity (delta Fisher's Z-score ketamine- placebo for each subject). 95% CI is indicated by dotted lines and linear regression straight line with functional equation in solid line.

The mean delta values of the clinical scores of PANSS and 5D-ASC are shown for ketamine and placebo separately in Table 5, all subitem values of the tested clinical scores were significantly increased by ketamine. Three significant correlations between a clinical symptom score and correlation changes were found (shown in Table 6): negative symptoms (PANSS) with ECN-L. calcarine fissure as well as with SN- R. calcarine fissure and VIR with DMN- L. superior parietal lobule. Only the correlation SN- R. calcarine fissure with negative symptoms (PANSS) survived more stringent Bonferroni correction. The correlation is shown in a scatter plot in Fig. 7. There is a positive correlation between functional connectivity and the clinical score. There was no significant correlation between shortened sessions lengths in ketamine condition and altered clinical symptom scores on group level, see Table 7.

**Table 5:** Seventeen subjects, comparison of clinical symptom scores and sub-items between ketamine and placebo condition with repeated measure t-tests. 5D-ASC subitem scores were divided by 10 for better visualization.

Parameter	Placebo	Ketamine	T	P
	mean (SD)	mean (SD)		
PANSS sum	0 (0)	41.71 (19.40)	8.6	<0.0001
PANSS positive	0 (0)	10.29 (6.65)	6.19	<0.0001
PANSS negative	0 (0)	8 (4.98)	6.42	<0.0001
OBN	1.88 (5.10)	104.8 (56.31)	7.47	<0.0001
DED	2.43 (5.11)	79.59 (47.22)	5.99	<0.0001
VRS	1.60 (2.93)	55.56 (41.07)	4.49	<0.0001
AUA	1.25 (2.39)	41.23 (39.30)	4.32	<0.0001
VIR	4.14 (7.54)	60.96 (25.76)	5.19	<0.0001

Abbreviations: “oceanic boundlessness” (OBN), “dread of ego dissolution” (DED), “visionary restructuralization” (VRS), “auditory alterations” (AUA) and “vigilance reduction” (VIR).

**Table 6:**
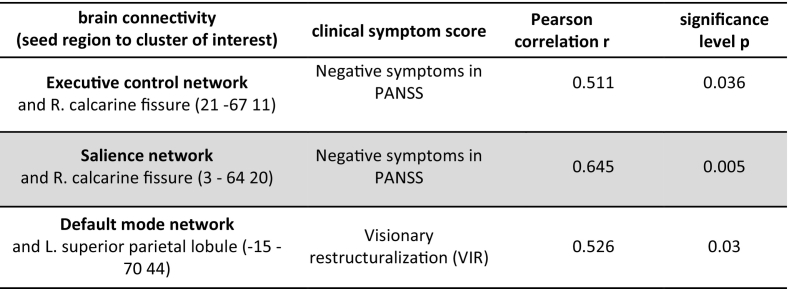
Correlation testing between brain connectivity and clinical symptom score by using bivariate Pearson correlation (p < 0.05). Grey background indicates result significant after Bonferroni correction.

**Table 7:** Seventeen subjects, group level correlation testing between delta of number of volumes (ketamine- placebo) and delta clinical symptom scores (ketamine- placebo) by using bivariate Pearson correlation (p < 0.05). No Bonferroni correction applied.

Parameter	Pearson correlation r	Significance level p
Positive symptoms	0.041	0.875
Negative symptoms	0.208	0.423
Sum	0.067	0.797
OBN	0.305	0.797
DED	0.224	0.388
VRS	0.053	0.84
AUA	0.0001	0.999
VIR	0.17	0.948

Abbreviations: “oceanic boundlessness” (OBN), “dread of ego dissolution” (DED), “visionary restructuralization” (VRS), “auditory alterations” (AUA) and “vigilance reduction” (VIR).

## 4 Discussion

In the present study, we investigated subanesthetic ketamine effects on four RS-fMRI networks in healthy subjects with an fMRI analysis which is comparable to the analysis approach used in a recent schizophrenia study (Woodward et al., 2011). Head motion, a major source on confounds in fMRI studies, wasn't significantly different between the placebo and ketamine condition. After correction for multiple testing, significant ketamine induced RS-fMRI signal changes remained with the ECN and SN. The ECN seed showed increased functional connectivity with resting network hubs, such as the anterior cingulum and the frontal gyrus. This can be interpreted as overall increased connectivity of the prefrontal cortex with other resting-state networks via these hubs. We hypothesize that this effect is caused by disinhibition of pyramidal neurons through blockade of NMDARs on GABAergic interneurons which are abundant in the prefrontal cortex (Dawson et al., 2013). Increased metabolic activity in the prefrontal cortex, shown in mice using 2-deoxyglucose based imaging after application of 0.31 mg/kg/h body weight ketamine, supports this theory (Dawson et al., 2013).

Other significant ketamine-induced RS-fMRI signal changes were the decreased connectivity of the ECN and SN seed with the calcarine fissure in the occipital lobe, which represents a part of the primary visual cortex (Griffis et al., 2016). As these regions are associated with attention and executive functions, we interpret the decreased connectivity with an area being involved in visual information processing as a sign of decreased visual perception in these subjects. Physiologically, vision is processed from the primary visual cortex via the temporal cortex (ventral stream) or parietal cortex (dorsal stream) (Van Polanena and Davarea, 2015). Therefore, functional connectivity between frontal areas, such as SN or ECN, is generally not directly expected. Due the localization of the calcarine fissure close to the skull, results are more prone to motion artifacts, (Havsteen et al., 2017) though significant differences of motion weren't found. Our finding could also be interpreted as impaired NMDAR-related neural transmission between certain brain areas, including an area of visual processing. We are not aware of any earlier study reporting a comparable finding for these two networks. However, taking a similar ROI-analysis approach, Niesters et al reported ketamine-induced decreased functional connectivity in the somatosensory network in a variety of regions including occipital cortex whereas no such decrease of functional connectivity was seen in other networks (Niesters et al., 2012). Notably, these authors also did not observe any positive or negative ketamine effect on the DMN, similar to our findings. This suggests that ketamine-induced changes may not be seen in every network and that the direction of ketamine effects might be dependent on the particular network of investigation. As our findings aren't correlated with increased positive symptoms, e.g. delusions and visual hallucinations, these observations require further investigation in future research.

The negative symptoms (PANSS) were positively correlated with the SN and calcarine fissure connectivity. Subjects with minimal difference between pre- and postsession PANSS score showed increased negative connectivity. This result could indicate increased inhibition of the visual cortex by ketamine, similar to the results by Yu et al. in chronic ketamine administration in monkeys (Yu et al., 2012). Due to the excitatory effects of ketamine on the interneurons in the frontal brain, this effect could be indirectly caused by activation of inhibitory neurons in another brain region projecting on the primary visual cortex. Increased negative symptoms, e.g. decreased reactivity to stimuli, could be signs of disrupted information processing in areas of attention, e.g. the SN. We hypothesize, the inhibition of the visual cortex- SN connectivity by ketamine reduces this troubled information processing by reducing input to the SN, therefore subjects with strongly negative effects on functional connectivity show decreased negative symptoms. This could be a sign that the effects of ketamine differ regionally from excitation to inhibition. The inhibition of connectivity of the ECN and SN with the occipital love in subjects with high negative symptom scores suggests that negative symptoms are caused by neuronal inhibition and are specific to parts of executive control and sensory areas. But this hypothesis requires further research on fMRI connectivity changes between subjects with high negative symptoms against low negative symptoms. In this study such an analysis couldn't be performed, due to a limited number of data sets. On the other hand, positive symptoms are likely caused by excitation/ loss of inhibition in different brain areas. Our analysis didn't reveal significant correlations between positive symptoms and altered connectivity, so positive symptoms may be caused by other brain regions, e.g. subcortical areas, or within region that didn't show significant alterations in our analysis, but could be affected nevertheless, like the DMN.

In contrast to Bonhomme et al., we didn't find a strong influence of ketamine on the DMN. Furthermore, we didn't find any anticorrelations in contrast to their publication. We hypothesize that the PCC seed in our study doesn't represent the whole DMN and additionally seed regions, e.g. the ACC, would have helped to analyze ketamine effects on the DMN more precisely, especially in the frontoparietal part. The finding that anticorrelation gets diminished in deeper sedation could be a reason it wasn't a finding of the present study. In our study, dosages and not the sedation levels were defined, therefore some subjects might have had a deeper level of sedation than others, causing the anticorrelations not to be significant on group level.

In recent RS-fMRI ketamine studies, whole-brain connectivity and region-specific analysis approaches were both used to investigate ketamine effects. In contrast to region-specific approaches, e.g. ROI-analysis, which was used in our study, whole-brain analysis doesn't require a priori-hypotheses about pharmacological effects like preselected seed regions. Recently, two studies were conducted to determine global ketamine effects on resting fMRI connectivity using a “degree of centrality”-analysis (Joules et al., 2015) and a “global-based connectivity” (GBC) (Driesen et al., 2013). Joules and Coworkers reported ketamine shifts brain connectivity from a cortical to a subcortical brain state with a decrease of connectivity of cortical hubs (Joules et al., 2015). In contrast, GBC-analysis showed ketamine-dependent increased global connectivity both in cortical and subcortical regions with no discrete clusters of increased GBC within this overall pattern. A number of regions identified by GBC in the ketamine condition predicted positive symptoms. Most notably, a correlation between increased connectivity in thalamus and striatum on one side with reduced negative symptoms of the PANSS on the other side was also observed (Driesen et al., 2013). In our study, global effects of ketamine weren't measured as we attempted to identify specific regions affected by drug challenge. However, increased connectivity, especially with the ECN, is consistent with the reported global effects. We found no significant correlations of ketamine-induced increase of connectivity with the PANSS positive symptoms scale. Reasons for the discrepant findings could be related to differences in the study designs. They administered a ketamine dose which was considerably higher compared to the dosage chosen in our study and therefore more likely to cause strong clinical effects. Nevertheless, the increase of positive and negative symptoms in our study were significantly larger than reported in the mentioned study. Apart from these study design considerations, our hypothesis-based approach with preselection of functional networks might have been too conservative to detect statistically significant correlations with the PANSS positive symptom score. Using cortical seeds exclusively may also account for our inability to detect ketamine-induced increases of subcortical connectivity being positively correlated with PANSS positive symptoms and negatively correlated with PANSS negative symptoms as reported by Driesen et al.

In drug research and development, ketamine is a widely used model substance to mimic clinical symptoms of acute schizophrenia in healthy subjects (Frohlich and Van Horn, 2014). However, the question is whether ketamine-induced changes of RS-fMRI can serve the purpose as a pharmaco-imaging model of schizophrenia. When comparing findings with the results of a schizophrenia study (Woodward et al., 2011), it is difficult to reconcile the findings in schizophrenia patients with our ketamine findings. Differences between both studies are particularly obvious in the DMN. In contrast to our findings, Woodward and colleagues showed an altered DMN resting network connectivity pattern in patients with schizophrenia, with functional connectivity of the DMN being increased involving several brain regions, e.g. including the middle frontal gyrus, inferior gyrus and middle temporal lobe. Another observation by them was a decreased DN and ECN functional connectivity in schizophrenia patients in contrast to the increased ECN connectivity in our study. The differences compared to the findings of Woodward et al can't be explained by different seed regions and could be explained by long-term effects of schizophrenia on connectivity compared to acute ketamine symptoms.

### 4.1 Limitations

Limitations of our study were the selection of male subjects, reducing the results of this study to being only applicable to half of the population. A large number of missing or prematurely terminated ketamine RS-fMRI sessions limited the number of usable data sets. Shortening the session to a similar length causes a selection bias since strong clinical ketamine effects (associated with e.g. excitement, hallucinations) were less likely to be included in this analysis. The experimental approach with assessment of clinical scores before and after each scan also did not allow to determine the exact onset of ketamine-induced clinical symptoms, likewise ketamine plasma concentration levels were not measured during our experiment. Furthermore, the dosage of ketamine infusion was rather low compared to other studies. Important vital signs, e.g. blood pressure, pO2-saturation and partial CO2-pressure were monitored but weren't available for our fMRI analysis. These data could have been useful for conclusions on the clinical effects of ketamine during the scan and for the differentiation between subjects with more clinical symptoms and lesser symptoms. Using a seed-to-voxel approach with predefined ROIs reduces the possibilities for discovering all ketamine induced changes and selection of seeds limited the findings to cortical areas exclusively. The extrapolation of intrinsic activity of resting-state network from single seeds may not necessarily be representative for the whole network. Additionally, RS-fMRI lacks cognitive specificity; therefore, finding significant drug effects in small data sets is impeded. Future research should address the question, whether these findings are robust in other experiment.

## 5 Conclusion

In summary, we found significant ketamine effects on two RS-fMRI functional networks (executive control network, salience network) with high accuracy. Among these, positive ketamine effects were found in the ECN, which is consistent with our hypothesis of increased functional connectivity of the prefrontal cortex. The clinical symptom scores 5D-ASC and PANSS also were significantly increased in ketamine condition. However, the major finding is decreased functional connectivity between SN and calcarine fissure being strongly correlated with negative symptoms. This suggests that this decreased functional connectivity might be a possible surrogate outcome biomarker for negative symptoms in future ketamine drug trials.
